# Trustworthy management in hospital settings: a systematic review

**DOI:** 10.1186/s12913-023-09610-5

**Published:** 2023-06-20

**Authors:** Andreea Isabela Varga, Ivan Spehar, Helge Skirbekk

**Affiliations:** 1 Department of Health Management and Health Economics, Institute of Health and Society, Medical Faculty, University of Oslo (UiO), P.O. Box 1089, Oslo, NO-0317 Norway; 2 Institute of Psychology, Oslo New University College, Oslo, Norway; 3 Department of Nursing and Health Promotion, Faculty of Health Sciences, Oslo Metropolitan University, Oslo, Norway

**Keywords:** Trust, Trustworthiness, Trust relations, Hospital, Healthcare professionals, Supervisor, Management, Leadership, Management roles, Systematic review

## Abstract

**Background:**

Trustful relationships play a vital role in successful organisations and well-functioning hospitals. While the trust relationship between patients and providers has been widely studied, trust relations between healthcare professionals and their supervisors have not been emphasised. A systematic literature review was conducted to map and provide an overview of the characteristics of trustworthy management in a hospital setting.

**Methods:**

We searched Web of Science, Embase, MEDLINE, APA PsycInfo, CINAHL, Scopus, EconLit, Taylor & Francis Online, SAGE Journals and Springer Link from database inception up until Aug 9, 2021. Empirical studies written in English undertaken in a hospital or similar setting and addressed trust relationships between healthcare professionals and their supervisors were included, without date restrictions. Records were independently screened for eligibility by two researchers. One researcher extracted the data and another one checked the correctness. A narrative approach, which involves textual and tabular summaries of findings, was undertaken in synthesising and analysing the data. Risk of bias was assessed independently by two researchers using two critical appraisal tools. Most of the included studies were assessed as acceptable, with some associated risk of bias.

**Results:**

Of 7414 records identified, 18 were included. 12 were quantitative papers and 6 were qualitative. The findings were conceptualised in two categories that were associated with trust in management, namely leadership behaviours and organisational factors. Most studies (n = 15) explored the former, while the rest (n = 3) additionally explored the latter. Leadership behaviours most commonly associated with employee’s trust in their supervisors include (a) different facets of ethical leadership, such as integrity, moral leadership and fairness; (b) caring for employee’s well-being conceptualised as benevolence, supportiveness and showing concern and (c) the manager’s availability measured as being accessible and approachable. Additionally, four studies found that leaders’ competence were related to perceptions of trust. Empowering work environments were most commonly associated with trust in management.

**Conclusions:**

Ethical leadership, caring for employees’ well-being, manager’s availability, competence and an empowering work environment are characteristics associated with trustworthy management. Future research could explore the interplay between leadership behaviours and organisational factors in eliciting trust in management.

**Supplementary Information:**

The online version contains supplementary material available at 10.1186/s12913-023-09610-5.

## Background

Trustful relationships between professionals are an important quality of both successful organisations and well-functioning hospitals [1, 2]. Professional workers in high-trust organisations are happier, more productive, have more energy, collaborate better, and are more loyal to their organisations than people working in low-trust companies [2]. Studies in hospital settings seem to indicate similar findings. In Taylor & al.’s [1] systematic review study of factors and strategies associated with high performing hospitals, trustful relationships was found to be one of the more important factors. High performing hospitals demonstrated respectful and valued relations between staff members [3, 4].

The phenomenon of trust has been widely studied. A commonly used definition is Mayer, Davis and Schoorman’s (1995) definition of trust as the “willingness of a party to be vulnerable to the action of another party based on the expectation that the other will perform a particular action important to the trustor, irrespective of the ability to monitor or control that other party” [5]. Within the healthcare sector, the published literature has explored many facets of trust, such as trust in healthcare in general [6–8], trust between patients and providers [9–11], trust between healthcare providers [12, 13] and trust between healthcare providers and their supervisors [14, 15].

Studies have showed that trust is important in relations between healthcare professionals and patients. Patient trust has an impact on patient satisfaction, adherence, and continued enrolment [16–19]. Trust is also highly important for the level of openness in communication between doctors and patient [20]. According to many theoretical approaches to the study of trust, a central aspect of trust relationships is the trustor’s lack of precautionary measures against the trustee [21–23]. Patients are vulnerable because of their illness, and the asymmetrical knowledge of medicine [24–26].

McCabe and Sambrook [27] studied the antecedents, attributes and consequences of trust between nurses and nurse managers. In terms of consequences, when trust was “high” there were positive outcomes such as professionalism, efficiency and a high quality patient care delivered; while the contexts where trust was low or lacking, led to negative effects such as conflict, absenteeism and turnover; reduced levels of teamwork, patient care quality, support, delegation and efficiency; and increased levels of work-related stress and surveillance [27].

These very different studies point in divergent directions. We understand that trust is often associated with positive outcomes for both patients and healthcare professionals. But we lack a systematic review of trust between healthcare providers and their supervisors. A handful of systematic literature reviews focused on patients’ trust in their healthcare providers [10, 28, 29], and one reviewed literature on healthcare professionals’ trust in patients [11]. In terms of trust relations between healthcare professionals and their supervisors, one systematic review explored how motivation is influenced by such relationships [13]. However, there is a lack in the overview of the published literature on what characterises this trust relationship between employees and their supervisors within a hospital setting.

Given this gap in knowledge, we aim to study the trust relationships, or lack thereof, between healthcare staff and their supervisors by conducting a systematic review that will map and provide an overview of the published literature on this topic. We want to study: 

What are the characteristics of trustworthy and/or untrustworthy management, be it culture of sharing, management style and tools, manager characteristics, etc; in a hospital or a similar setting such as wards or large general/family practices?

## Methods

### Search strategy

Seven databases (Web of Science, Embase, MEDLINE, APA PsycInfo, CINAHL, Scopus and EconLit) and three publisher platforms (Taylor & Francis Online, SAGE Journals and Springer Link) were searched systematically to find eligible records. These sources were searched based on the relevance of the fields they covered to the subject of this review, such as medicine, social sciences, nursing and allied health and healthcare policy and management.

The search strategy used to identify relevant records was developed over the course of nine months. The final structure of the search strategy was the product of an iterative process which involved testing of different variations of the search strategy, and discussions among the authors and experts on systematic reviews. The input of an expert in running searches, a university librarian, was sought in order to reach a sound search strategy.

The search strategy has three components and has the following structure: 1) “hospital(s)” OR “ward(s)” AND 2) “health care professional(s)” OR “doctor(s)” OR “nurse(s)” OR “leader(s)” OR “manager(s)” AND 3) “trust” OR “reliance” OR “credibility”. The first component filters by the setting this review is focused on, namely hospitals. The second component establishes the actors/stakeholders within a hospital and is captured by the terms listed above and their synonyms. The third component represents the interaction or relationship between the actors and is linked to the search strategy with a proximity operator. Proximity operators were also used for some of the terms in the second component of the search strategy in order to make the strategy more specific, like “(healthcare NEAR/x professional$)”. Where applicable, the searches were limited to English language.

The detailed search strategy can be found in an additional file [see Additional file 1]. The final search was carried out on the 9th of August 2021.

### Eligibility criteria

For records to be included in this review, several inclusion criteria were applied. Firstly, in terms of context and participants, eligible studies had to be undertaken in a hospital setting or similar settings where healthcare professionals and managers are present and patients are being treated. Secondly, related to topic, studies should have addressed and explored aspects relevant to the relationship of trust / trustworthiness of subordinates with their higher-ups. Thirdly, eligible records should be of empirical nature. Initially there was no exclusion based on study design; this criterion was later changed in the full-text screening review, as systematic reviews were excluded. However, this criterion remained broad as this review aimed to identify studies of qualitative, quantitative and mixed designs. This was motivated by our purpose to capture, on one hand, the objectiveness that quantitative studies offer on the topic, and on the other to capture the in-depth understanding that qualitative studies provide. Lastly, articles should be written in English. No limit on the year of publication was imposed.

### Record selection

**Fig. 1:**
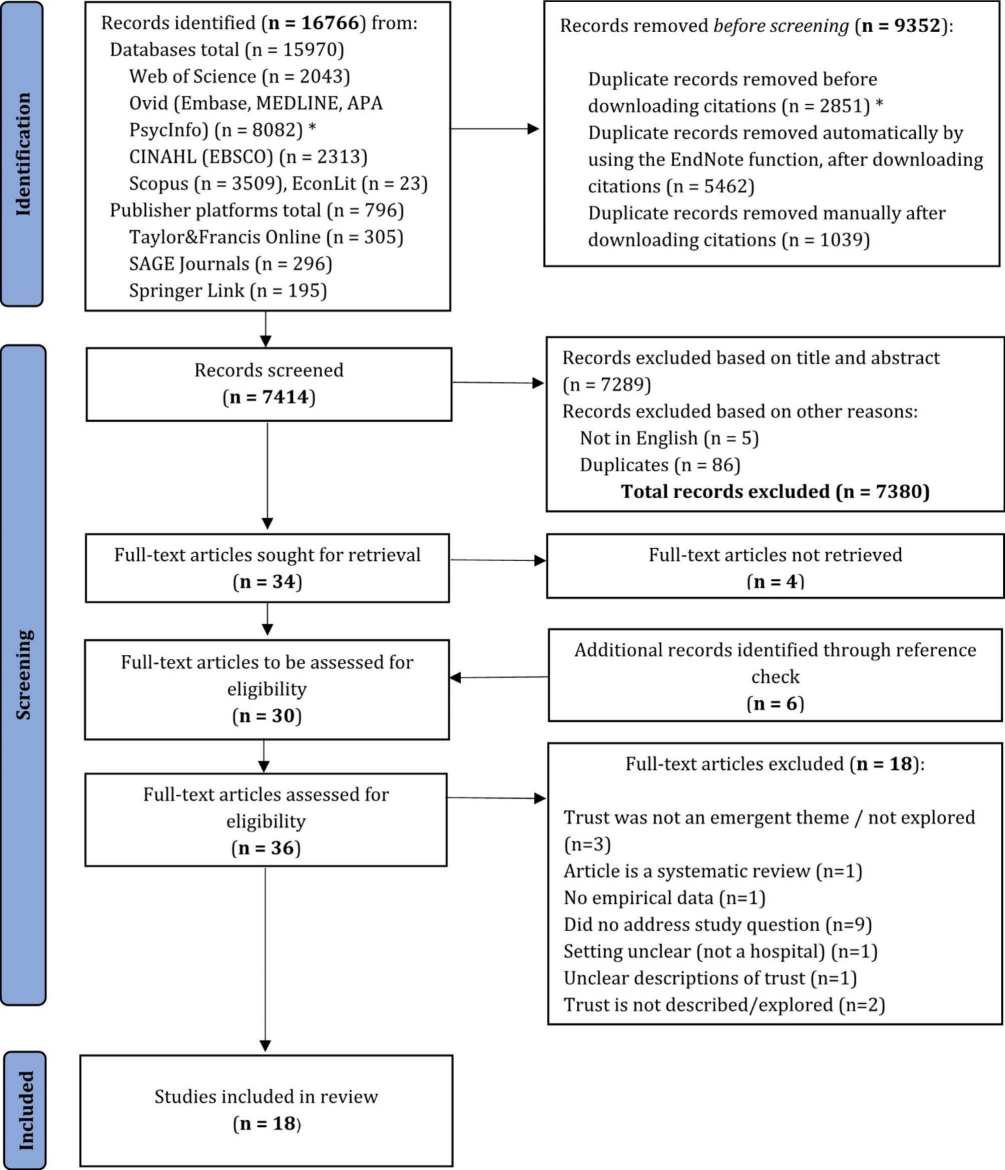
Flow diagram. * The Ovid platform provided the option of removing duplicates from the records identified before downloading the citations, as the search was performed in multiple databases at once. Adapted from Page, McKenzie [30]

The processes of identification, screening and inclusion are depicted in Fig. 1. Flow diagram.

The search resulted in a total of 16,766 records, 15,970 from databases searches and 796 from publisher platforms. Then 9352 duplicate records were removed before the screening process. More specifically, 2851 duplicates were removed before the citations were downloaded. These come from the search conducted in the Ovid platform, which allowed deduplication for the search performed in multiple databases (Embase, MEDLINE and APA PsycInfo) at once.

After the citations were downloaded and imported in the EndNote X9 reference manager, 5462 duplicates were automatically identified by the reference manager. An additional 1039 duplicates were identified manually and removed. Thus, after all duplicates were removed, a total of 7414 records were screened.

The first half and second half were independently screened by two researchers. IS and AIV screened the first half, while HS and AIV screened the second half. The screening comprised of scanning the titles and abstracts. A total of 7380 records were removed; 7289 did not meet the inclusion criteria, based on title and abstract, 5 records were not written in English and an additional 86 duplicate records were found. The 47 remaining publications were discussed by all three researchers, with a focus on the ones that we were in disagreement over. The discussions resulted in 2 records out of the 15 previously agreed upon to be excluded and 21 publications out of the 32 were agreed to be included in the full-text review. Thus, a total of 34 publications were sought for retrieval.

30 records were retrieved. For four records, a full-text version could not be retrieved. The authors of these papers were contacted through Research Gate, but no reply was given. An additional number of 6 papers were identified through reference check of the included records. These were retrieved, and a final number of 36 full-text articles were assessed for eligibility.

The full-text review was performed independently by all authors, and 18 articles met the inclusion criteria. The rest (n = 18) were excluded based on the reasons listed in Fig. 1. The list of the excluded papers can be found under Additional file 2.

### Data analysis and synthesis

Given the descriptive nature of this review’s research question, and the inclusion of papers with different research designs (both qualitative and quantitative), a narrative approach to data analysis and synthesis was adopted. This entailed developing textual and tabular summaries of findings, which were then used to synthesise the findings under two separate sets of factors.

Data extraction was performed by one researcher (AIV) and checked for correctness by another (IS), and comprised of three categories. The first one relates to details about the included studies: author(s), year of publication, aim(s), methodology (design, setting, participants and sample, instrument and measured concepts, data analysis) and country. The detailed summary of included studies can be found under Additional file 3. The second category comprises of results relevant to the research question and the concept of trust extracted from quantitative studies, such as hypotheses and whether they were supported or not. The extracted data for the second category is available under Additional file 4. And the third category similarly gathered results pertinent to the research question from qualitative studies, such as themes identified by the authors of the included studies and their interpretations of supporting evidence quoted from interviews. The extracted data included in this category can be found under Additional file 5.

Once all the data was extracted, based on his experience in the field of leadership, management and organisations, IS observed patterns in the results. More specifically, characteristics of trustworthy management were noticed to fit under two categories, namely leadership behaviours and organisational factors. IS then summarised and categorised the results into the two classifications. These summaries were presented and discussed with the two other authors during the process. All authors agreed that the summaries were representative of the original findings. The summaries were presented as tables in the results section.

The results section firstly described the study characteristics, then laid out common aspects identified between the qualitative and quantitative studies included in the review. The common aspects related to trust and ethics, trust and well-being, trust and availability and trust and competence. Aspects not common between the quantitative and qualitative studies were presented separately.

All included studies were critically appraised by two researchers. The qualitative studies (n = 6) were assessed by HS and AIV using the JBI Critical appraisal checklist for qualitative research [31] and the quantitative studies were appraised by IS and AIV using an adapted checklist by the National Institute for Health and Clinical Excellence (NICE) for a questionnaire study [32]. The NICE checklist did not provide response options, and in order to be consistent, we decided to use the ones from the JBI checklist (Yes, No, Unclear and Not applicable). The overall appraisal scale published by Roever [33] was used to rate the overall methodological quality of the studies included in this review. The results of the critical appraisal were used to provide an overall picture over the quality of the included studies and to determine whether there were any papers of poor quality, with significant flaws that would determine their exclusion from this review.

## Results

### Research methods, setting and participants, journals and countries

Tables 1 and 2 lay out the summaries of the quantitative and qualitative included studies in a concise manner. The majority of the studies used a quantitative research design (n = 12), in which surveys (n = 5) and questionnaires (n = 7) were self-administered. With two exceptions, studies (n = 11) collected the data at one point in time. The first exception is a study in which the data was gathered sequentially; with two weeks between the collection of demographic, independent and dependent variables. And the second exception is a study that had a three-week follow-up, but no details are presented. The rest of the included studies had a qualitative design (n = 6); two of which solely collected data through interviews, while the rest (n = 4) used a combination of interviews, focus groups, document reviews or observations such as participant observations, facility audits and research memos.

**Table 1:** Summary of included quantitative studies

Author(s) (Year)	Setting	Participants	Measured concepts	Data analysis	Findings		Country
					Leadership behaviours associated with trust in management	Organisational factors associated with trust in management	
Araujo and Figueiredo [43]	Private hospitals (N = 5)	Nurses (N = 171) and nurse technicians (N = 274)	Internal climate dimensions (trust being one of them) and positive attitudes and behaviours	Multivariate techniques factor analysis, multiple linear regressions	Making employee’s job easier, caring about their well-being at work, being accessible and open to dialogue.	Ability to participate in hospital decisions. Having individual rights respected.	Brazil
Bai, Lu [34]	Large-scale general hospitals (N = 15)	General employees (N = 2365) and department leaders (N = 270)	Paternalistic leadership, personal initiative and general employees’ affective trust in their direct leaders.	Multilevel modelling analyses – hierarchical linear modelling	Moral leadership Authoritarian leadership (negative relation)	N/A	China
Bobbio, Bellan [44]	Public general hospital (N = 1)	Nursing staff (N = 273)	Empowering leadership, perceived organisational support, trust in leader (nurse manager) and in organisation	Pearson’s correlation coefficients and structural equation modelling (SEM)	Empowering leadership.	Trust in the organisation.	Italy
Bobbio and Manganelli [45]	Public general hospital (N = 2)	General nursing staff (N1 = 371, N2 = 340)	Servant leadership, perceived organisational support, trust in leader (nurse manager) and in organisation	Correlational analyses and SEM	Servant leadership	Perceived organisational support.	Italy
Coxen, van der Vaart [35]	Hospitals and/or clinics (N = 27)	Administrative, management, specialist and other employees (N = 633)	Authentic leadership and workplace trust (trust in the organisation, trust in the immediate supervisor and trust in co-workers).	SEM	Authentic leadership	N/A	South Africa
da Costa Freire and Azevedo [47]	Public hospitals (N = N/A)	Professionals from various nursing services (N = 189)	Background of empowerment and perceptions of trustworthiness of supervisor	Descriptive statistics and SEM.	N/A	Empowering work context.	Portugal
Enwereuzor, Adeyemi [36]	Hospitals (N = 10)	Staff nurses (N = 237)	Ethical leadership and trust in leaders (ward/unit leader or immediate supervisor)	Descriptive statistics, correlations and ordinary least squares regression-based path analysis (using bootstrapped samples)	Ethical leadership	N/A	Nigeria
Fleig-Palmer, Rathert [37]	Acute care hospital and associate clinics	Clinical and non-clinical workers (N = 315)	Mentoring function, trustworthiness factors and trust in a mentor or manager	Common method variance analysis	Mentoring behaviours (mediated through ability, integrity and benevolence).	N/A	USA
Laschinger, Finegan [48]	Urban tertiary care hospitals (N = N/A)	Nurses (N = 412)	Work empowerment, formal power, informal power and interpersonal trust at work (more specifically, faith and confidence in the intentions of peers and managers)	SEM	N/A	Empowering work context.	Canada
Simha and Stachowicz-Stanusch [49]	Hospitals (N = 7)	Hospital administrators and management personnel (N = 178)	Ethical climate, trust in supervisor, trust in organisations	Factor analyses and SEM	N/A	Benevolent-local climates. Egoistic-local climates (negative relation).	Poland
Stander, de Beer [39]	Public hospitals and clinics (N = 27)	Management, specialist, administrative and other employees (N = 633)	Authentic leadership and trust in the organisation	SEM	Authentic leadership*		South Africa
Wong and Cummings [46]	Cancer treatment facilities (N = 17)	Clinical (N = 147) and non-clinical employees. (N = 188)	Leadership practices, perceptions of work-life and emotional health and well-being.	SEM	Supportiveness (significant indirect effect). Relational transparency (for a non-clinical sample).	Salary, workload, administrative support.	Canada

N/A = not applicable, SEM = structural equation modelling *Authentic leadership was associated with trust in the organisation. The authors did not measure trust in management directly

**Table 2:** Summary of included qualitative studies

Author(s) (Year)	Setting	Participants	Measured concepts	Data analysis	Findings*	Country
					Leadership behaviours associated with trust in management	
Cregård and Eriksson [15]	Hospitals (N = 3)	Interviews with part-time physician-managers (N = 8) and full-time nurse-managers (N = 8), focus groups (N = 6) and a post-data saturation focus group	Perceptions of perceptions were investigated. Physician-managers were asked about their perception of other physicians’ trust in them; and nurse-managers were asked how they perceive physicians’ trust in physician-managers. The post-data saturation focus group addressed one main question: “What does diminished trust mean and does it matter?” (p.286)	An analytical model based on theories of trust was used to analyse the data	Ability, benevolence and integrity. Difficulties in combining managerial and medical role (negative relation).	Sweden
Freysteinson, Celia [38]	Hospitals (N = 11)	Individuals with leadership experience (N = 28) were interviewed	Semi-structured interview questions related to nursing leaders’ feelings, decisions, motives and self-talk during a crisis (pandemic)	Naïve reading, structural explanation and phenomenological interpretation.	Face-to-face interaction. Transparency.	USA
McCabe and Sambrook [27] **	Hospitals (N = 2)	Interviews with nurses and nurse managers (N = 39) from an acute and a community hospital.	Semi-structured, open-ended questions related to “*(1)* how participants conceptualised trust and the level of trust within their working environment, *(2)* the characteristics and attributes of trust and trustworthy managers and *(3)* the consequences of low trust” (p. 819)	Concept analysis, thematic analysis	Leadership, professionalism, honest and clear communication, confidentiality.	UK
Stasiulis, Gibson [40]	Early psychosis intervention clinic (EPI) (N = 1)	Interviews (N = 27) with staff, young people and family members, participant observations and document reviews.	In-depth interviews were guided by “questions that focused on obtaining accounts of participants’ everyday activities in the clinic” (p.3)	Reading and mapping	Servant leadership. Including staff in decision making.	Canada
Topp and Chipukuma [41]	Primary health centres (N = 4)	In-depth interviews with healthcare workers (N = 60) and key-informants (N = 14), semi-structured interviews with patients (N = 180), facility audits, observations and research memos.	The questions posed in the interviews were aimed “to elicit detailed descriptions of interactions among and between staff and patients to provide insight into whether and why trust may be present in certain relationships” (p.195)	Deductive and inductive analysis	Fairness and consistency. Problem-solving capacity. Communication.	Zambia
Weaver, Lindgren [42]	Hospitals (N = 37)	Staff nurses (N = 39) participated in focus groups, and administrative supervisors (N = 30) were interviewed.	The participants were asked about the practices of administrative supervisors	An inductive, systematic and iterative analysis strategy was employed	Being friendly, approachable and “a people person”. Being unapproachable (negative relation).	USA

* No papers, except one, discussed organisational factors associated with trust in management. ** The authors found that antecedents of trust converged mainly on organisational factors (work environment, communication)

In terms of setting, studies took place in hospitals (n = 12), hospitals and clinics (n = 3), cancer treatment facilities (n = 1), primary health centres that include inpatient departments (n = 1) and an early psychosis intervention (EPI) clinic (n = 1). Some of the quantitative studies were conducted from the perspective of healthcare employees (nurses and nursing staff) (n = 6), other studies focused on the perspective of employees in management, specialist or administrative positions (n = 3) and three studies included both perspectives. Similarly, one qualitative study captured the perspective of healthcare workers and key-informants, two studies described the management perspective and the rest (n = 3) included both.

Several studies (n = 6) were published in journals that include the healthcare field, such as leadership and management-oriented journals (n = 2), human resources journals (n = 2), industrial psychology (n = 1) and social behaviour (n = 1). While the rest (n = 12) were published in journals covering the healthcare area specifically. The journals were related to management (n = 2), policy and planning (n = 1), leadership (n = 3) and social science and medicine (n = 1) in a general sense, while a handful of studies were published in journals related to nursing specifically (administration and management) (n = 6).

Most of the studies were conducted in the Americas, namely USA (n = 3), Canada (n = 3) and Brazil (n = 1). Then other studies were conducted in European countries, more specifically Italy (n = 2), Poland (n = 1), Portugal (n = 1) and Sweden (n = 1) and the UK (n = 1). Four studies were conducted in countries on the African continent such as South Africa (n = 2), Nigeria (n = 1) and Zambia (n = 1); and one study was conducted in China.

### Common aspects

This section firstly describes the two categories that were found to be associated with trust in management, namely leadership behaviours and organisational factors. Then, under the first category, four common aspects across both quantitative and qualitative papers are presented and can be seen under Table 3.

**Table 3:** Common aspects between quantitative and qualitative studies related to leadership behaviours associated with trust

Author(s) (Year)	Ethics	Well-being	Availability	Competence	
Araujo and Figueiredo [43]*	N/A	One of the items used to measure trust was: “The superiors care about my well-being at work”.	Another item used to measure trust was: “My superiors are accessible and open to dialogue”.	N/A	
Bai, Lu [34]*	Trust was positively related to direct leaders’ moral leadership.	N/A	N/A	N/A	
Bobbio, Bellan [44]*	N/A	Showing concern (which was one if the five dimensions of empowering leadership) was positively correlated with trust in the leader.	N/A	N/A	
Cregård and Eriksson [15]**	Integrity element of trust.	Benevolence element of trust.	N/A	Ability element of trust.	
Enwereuzor, Adeyemi [36]*	Staff nurses’ trust in their ward/unit leader or immediate supervisor is positively related with ethical leadership	N/A	N/A	N/A	
Fleig-Palmer, Rathert [37]*	The relationship between interpersonal mentoring behaviours and trust was mediated by integrity (element of trust).	The relationship between interpersonal mentoring behaviours and trust was mediated by benevolence (element of trust).	N/A	The relationship between informational mentoring behaviours and trust was mediated by ability (element of trust).	
Freysteinson, Celia [38]**	N/A	N/A	Face-to-face interaction crucial to earning trust of the employees and being transparent can increase trust.	N/A	
McCabe and Sambrook [27]**	N/A	N/A	Nurse managers were more likely to be trusted by nurses if they were considered accessible, approachable and involved.	“Professional competence”, alongside other behaviours, was considered an attribute of “’trusted’ line-managers and nurses”.	
Topp and Chipukuma [41]**	Perceptions of supervisors being unfair or inconsistent contributed to weak trust from healthcare workers.	N/A	N/A	Perceptions of lack of problem-solving capacity of supervisors contributed to weak trust from healthcare workers.	
Wong and Cummings [46]*	N/A	For the clinical sample of employees, supportiveness had a significant indirect effect on trust in management.	For the non-clinical sample of employees, relational transparency had a direct and significant effect on perceptions of trust in management.	N/A	

* = quantitative study design; ** = qualitative study design; N/A = not applicable

Most of the studies explored leadership behaviours associated with trust in management only (n = 10)[15, 34–42]. While five studies described characteristics related to both leadership behaviours and organisational factors that were associated with trust in management [27, 43–46]. Additionally, three studies explored organisational factors exclusively [47–49].

The common aspects are: trust and ethics, trust and well-being, trust and availability and trust and competence and were informed by the following leadership behaviours most commonly related to employees’ trust in their supervisor: different facets of ethical leadership (n = 5), caring for employees’ well-being (n = 5), the manager’s availability (n = 4) and leaders’ competence (n = 4). Each aspect and the studies that informed them are presented below.

### Trust and ethics

This first aspect was informed by five studies, two qualitative papers [15, 41] and three quantitative studies [34, 36, 37]. The different aspects of ethical leadership that were addressed included integrity [15, 37], moral leadership [34], fairness [41], and ethical leadership, specifically [36].

Cregård and Eriksson investigated physician-managers’ and nurse-managers’ perceptions of other physicians’ trust in them; and revealed that trust is strengthened by physician-managers’ understanding of “healthcare issues from various perspectives”, but can decrease when physician-managers are “unable to prioritize both managerial and medical issues” or “fulfil professional demands” ([15], Table I. p.287).

In Topp and Chipukuma’s interview study [41], healthcare workers perceived their supervisors in charge of overall or departmental sites to be unfair and inconsistent, for example when selecting staff for workshops or trainings; which contributed to weak trust.

Results from survey studies showed that employee’s affective trust in their direct leaders was positively related to their moral leadership [34]; and that staff nurses’ trust in their ward/unit leader or immediate supervisor was in a positive relationship with ethical leadership [36].

### Trust and well-being

One qualitative study [15] and four survey studies [37, 43, 44, 46] informed the second aspect. Caring for employees’ well-being included measures of benevolence [15, 37], supportiveness [46] and showing concern (one of five dimensions of empowering leadership in Bobbio et al.’s [44] study). In Araujo and Figueiredo’s study, trust was measured with five items, including: “The superiors care about my well-being at work” ([43], Table II.).

The qualitative study informing this aspect showcases that physician- and nurse-managers perceive that other physician’s trust in them is increased when the physician-manager shows care towards “patients, colleagues and other healthcare professionals” ([15], Table I. p.287).

In Wong and Cummings’s study [46], clinical (such as nurses, pharmacists, doctors and other professionals) and non-clinical employees (administrative, support and research staff) completed a survey with regards to trust in management; and the results were reported separately for the two samples. Supportiveness, as part of the leadership behaviour latent concept developed for the model that was tested in the study, had a significant indirect effect on trust in management among the clinical sample of employees ([46], p.14).

### Trust and availability

This third aspect was developed based on two qualitative papers [27, 38] and two quantitative papers [43, 46]. The manager’s availability was measured as being accessible [43] and approachable [46]. McCabe and Sambrook [27] found that nurse managers who were considered accessible, approachable and involved were more likely to be trusted by nurses. The opposite was true for managers who were “perceived as ‘inaccessible’, ‘removed’ or those managers higher up within the organisational hierarchy” ([27], p. 821). In Freysteinson et al.’s [38] study, availability relates to leaders’ efforts to maintain a visible and accessible leadership presence (with emphasis on face-to-face interaction with the staff).

In Araujo and Figueiredo’s study, another one of the five items that measured trust relates to: “My superiors are accessible and open to dialogue” ([43], Table II.). In Wong and Cummings’s study [46], another of the leadership behaviours, relational transparency, had a direct and significant influence on perceptions of trust in management, but only among the non-clinical sample of employees. There were no other direct significant effects between leadership behaviours and trust in management ([46], p. 14 and 16); making this study the only one included in this review that found mixed or no results for relationships between leadership behaviours and trust.

### Trust and competence

Three qualitative papers [15, 27, 41] and one quantitative study [37] informed this last aspect. The studies found that leaders’ competence, in terms of knowledge [37], medical competence [15] and decision making skills [27, 41], were related to perceptions of trust.

The physician-manager’s medical competence, on one side, was deemed “valuable when managerial healthcare decisions are required” and the participants (physician- and nurse-managers) perceived this as a factor that increased trust in the physician-manager ([15], Table I. p.287). On the other side, the participants also perceived that “physician-managers should have extensive involvement in medical practice” in order to maintain competence in daily medical work ([15], Table I. p.287).

### Organisational factors

One qualitative study [27] and seven quantitative studies [43–49] studied organisational factors associated with trust. Work environments in which employees experienced empowerment (n = 4) [43, 45, 47, 48] were most commonly associated with trust in management. Salary, workload and administrative support was also related to trust in one study [46]. In the qualitative study [27], the authors found that antecedents of trust converged mainly on organisational factors such as immediate work environment, communication systems and new management practices.

### Quantitative studies

The quantitative studies had different conceptualisations and measures of trust. Some studies measured trust as a one-dimensional concept, e.g. “trust in leader” [36] and “trust in supervisor” [49]. Other studies measured trust as a multi-dimensional concept. For example, in da Costa Freire and Azevedo’s [47] study, trust was operationalised as “perceptions of trustworthiness in the supervisor”, and measured on three dimensions (integrity, benevolence and ability). Laschinger, Finegan [48] separated trust into subscales measuring faith in the intentions of managers and confidence in managers’ actions. One study [43] conceptualised trust as one of nine dimensions related to internal climate at work.

Variations in the type of trust relationships investigated were also observed. For example, Bai et al. [34] studied general employees’ affective trust in their direct leaders. Bobbio et al. [44] and Bobbio and Manganelli [45] focused on nursing staff’s trust in leader (nurse manager in this case) and trust in the organisation; and similarly, another study [36] specified that nursing staff’s trust in leaders was understood as trust in their ward/unit leader or immediate supervisor. Additionally, one study [35] investigated workplace trust which was comprised of trust in organisation, trust in immediate supervisor and trust in co-workers.

### Qualitative studies

Among the six qualitative studies, four studies explored trust explicitly in the research aim [15, 27, 40, 41], while two studies identified trust as an emerging factor in the data analysis [38, 42].

In two of the qualitative studies [15, 38], trust was explored through managers’ own perspectives. Cregard and Eriksson [15] interviewed and conducted focus groups with physician managers and nurse-managers, with the aim of exploring trust in relation to physicians’ dual roles as managers and clinicians. According to the managers, aspects related to competence, benevolence, and integrity could influence physician employees’ trust in physician-managers. Difficulties related to combining the managerial and medical role was also described as a common reason for decreased trust. Freysteinson and colleagues [38] interviewed nursing leaders in American hospitals about their leadership experiences under the COVID-19 pandemic. The authors describe how the leaders became aware of face-to-face interaction as crucial to earning the trust of the employees, and that “leaders felt transparency increased trust” (p.1539). While the findings from these two studies were gathered from the lens of managers themselves, they are consistent with findings from other studies in our review.

### Critical appraisal

Table 4 presents the assessment of risk of bias for each paper included in the review.

**Table 4:** Critical appraisal of included studies

Author(s) (Year)	Quality Rating
Araujo and Figueiredo [43] *	**Acceptable (+)**
Bai, Lu [34] *	**Acceptable (+)**
Bobbio, Bellan [44] *	**Acceptable (+)**
Bobbio and Manganelli [45] *	**High (++)**
Coxen, van der Vaart [35] *	**Acceptable (+)**
Cregård and Eriksson [15] **	**Acceptable (+) / Low (-)**
da Costa Freire and Azevedo [47] *	**Acceptable (+)**
Enwereuzor, Adeyemi [36] *	**Acceptable (+)**
Fleig-Palmer, Rathert [37] *	**Acceptable (+)**
Freysteinson, Celia [38] **	**Acceptable (+)**
Laschinger, Finegan [48] *	**High (++)**
McCabe and Sambrook [27] **	**Acceptable (+)**
Simha and Stachowicz-Stanusch [49] *	**Acceptable (+)**
Stander, de Beer [39] *	**Acceptable (+)**
Stasiulis, Gibson [40] **	**Acceptable (+)**
Topp and Chipukuma [41] **	**High (++)**
Weaver, Lindgren [42] **	**Acceptable (+)**
Wong and Cummings [46] *	**Acceptable (+)**

* NICE critical appraisal checklist for a questionnaire study. ** JBI critical appraisal checklist for qualitative research. (++) = high quality (majority of criteria met, little or no risk of bias); (+) = acceptable (most criteria met, some flaws in the study with an associated risk of bias); (-) = low quality (either most criteria not met, or significant flaws relating to key aspects of study design) and (0) = reject (poor quality study with significant flaw, wrong study type, not relevant to guideline). Rating scale from: [33]

Out of the six qualitative papers assessed, most of them (n = 4) were rated as acceptable; while one paper was rated between acceptable and low quality and one paper as high quality. The majority of the quantitative papers (n = 10) were rated as acceptable and the rest (n = 2) were rated as high quality. Thus, most papers included in this study were assessed as acceptable. No study was excluded based on quality, as none were rated as poor (0).

## Discussion

### Summary of findings

This systematic literature review aimed to provide an overview of the published literature over the characteristics of the trust relationship between employees and their supervisors within a hospital setting. Based on the included studies, these characteristics were categorised under two aspects: leadership behaviours and organisational factors associated with trust in management. Most studies explored leadership behaviours, and thus some common aspects emerged between the qualitative and quantitative papers. The common aspects are: trust and ethics, trust and well-being, trust and availability and trust and competence. These are discussed below.

### Trust and ethics

Five included articles emphasised different aspects of ethical leadership for trust relationships to grow between employee and manager. Integrity, moral leadership, fairness and ethical leadership are mentioned specifically. In clinical studies on relationships between healthcare professionals and patients, it is more common to thematise reciprocity and “being taken seriously as a human being” [20]. Brown [50] has claimed that doctors’ standing as caring and competent now depends to a great degree on communication and involvement with the patient before trust can be earned. Showing reciprocal humanity creates common ground with the patient, and this review shows that similar effects play a role between leader and healthcare professionals.

Studies from other industries have also marked the impact ethical leadership has on trust in leader. For example, Newman et al. [51] showed that in a sample of n = 184 pairs of employees-supervisors from three Chinese firms, ethical leadership lead to higher levels of trust in leader (both cognitive and affective). Similarly, Dadhich and Bhal [52] found that ethical leadership predicted affective and cognitive trust in a sample of post-graduate engineering students in India.

### Trust and well-being

Several included studies showed a connection between managers’ care for the employees’ well-being and trust relationships. Being available when concerns are voiced, and listening to employees’ worries is important. A survey study on 107 white-collar employees working in various organisations in Malaysia [53] highlighted that when employees perceived their supervisor to show benevolence, integrity and ability, trust in them was predicted both directly and indirectly. Studies on the trust relationship between healthcare professionals and patients emphasise this characteristic even more clearly, as many studies have focused on how trust is built [20], and we can see some similarities to how trust is built between healthcare staff and managers. E.g., Skirbekk & al. have shown how relationships between healthcare professionals and patients based on “open mandates of trust” are more resilient [19]. The findings from the studies included in our study show that managers’ care for employees’ well-being lead to more caring and empowering trust relationships.

### Trust and availability

Manager’s availability was another leadership characteristic associated with trust in management, as shown by four papers included in this review; and had to do with managers being perceived as accessible and approachable. While there are few studies directly exploring the relationship between a supervisor’s availability and employees’ trust towards the supervisor, some studies from other organisational contexts have indicated that a supervisor’s availability might improve the quality of relations between supervisors and employees, both in physical [54] and remote work settings [55].

### Trust and competence

Four included studies found the leaders’ competence to be an important characteristic for trust relationships. Employees need to be assured that the leaders know what they are doing, or at least that they have a plan for how the hospital should be run. Similarly, Manderson and Warren [56] have shown how competence is often the most important dimension of trust relations with healthcare professionals. Studies on the doctor-patient relationship in different medical contexts have shown that the better a patient feels informed about the treatment process, the greater trust he or she will experience [57–60]. This trust in competence makes it possible for the patients to bridge the knowledge gap [24] through a “leap of faith” [25, 26]. There might be a similar “leap of faith” by health professionals towards their supervisors. Employees can rarely be expected to have knowledge on how hospitals should be run, but it is important for them to be able to trust that the leaders have this competence.

In terms of supervisors’ trustworthiness and competence, hospitals and related settings might place emphasis both on managerial and clinical competence. Studies of healthcare managers have found that doctors in management positions attempt to maintain their clinical competence. For example, Spehar & al. [61] found that Norwegian doctors in management positions in hospitals placed importance on “being perceived as a competent clinician in order to be taken seriously by the medical staff.“ The authors also found that clinical knowledge was important for “winning” arguments with the staff. This is in line with arguments by other authors on how doctors in management seek to maintain their clinical knowledge in order to sustain legitimacy among their staff, especially their professional colleagues [62, 63].

### Trust and culture

Studies have shown that there might be cultural differences in leader expectations and trust. Indeed, words such as «paternalistic», «feminine» and «masculine» are sometimes used to differentiate cultural expectations towards management [64, 65]. For example, employees in Western countries might expect a more «feminine», or empowering leadership style, whereas employees in Asian countries might expect a more paternalistic leadership style [66]. But studies have also shown similarities in expectations across different countries. For example, most employees want managers who are perceived as inspirational, competent and fair [67].

We have not observed explicit cultural differences in our included studies in terms of trust, although the number of studies included in our analysis might not be conducive to a comprehensive comparison of cultural differences. However, the study by Bai et al. [34], included in our study, found that authoritarian leadership of direct leaders had positive impacts on employees’ personal initiative. We can therefore not rule out that cultural differences might influence perceptions of trustworthiness.

### Methodological considerations

The fact that only one author extracted the data and no standardised data extraction form was used, could pose as a risk of error. This risk was reduced, as another author checked the correctness of the extracted data. Another drawback of this systematic literature review is that it was not registered and a formal review protocol was not used in guiding how this review was conducted. However, we did follow strict guidelines developed throughout years of experience and discussions with experienced reviewers. The expert knowledge of a librarian was also sought in the process of developing the search strategy. We also discussed conducting a more in-depth synthesis of the 6 qualitative papers, but we decided against it since we found the research questions in the included studies were not homogenous enough. This might be considered a missed opportunity.

### Quality of the included papers

14 of the 18 included articles have an acceptable quality. According to the rating scale we used [33], this means that most criteria were met but there are “some flaws in the study with an associated risk of bias”. For the qualitative studies rated as acceptable (n = 4), the associated risk of bias mostly arises from studies not locating the researcher culturally or theoretically, and not addressing the influence of the researcher on the research. For the quantitative studies rated as acceptable (n = 10), the associated risk of bias arose mostly from studies being unclear regarding whether the sampling frame was sufficiently large and representative; and somewhat from studies not discussing potential response biases. One qualitative paper was evaluated as having a quality between acceptable and low. An associated risk of bias stemmed from the study not locating the researcher culturally or theoretically and not discussing his/her influence on the research. The reason for leaning towards rating this paper low quality is the study failing to provide a statement on whether ethical approval by an appropriate body was granted.

Although the quality of the included quantitative papers was acceptable, and high in two cases, the use of surveys and questionnaires to capture an abstract concept such as trust can be viewed as a limitation. However, claims for the validity and reliability of the instruments used have been made and were justified in all papers, except for three, where the claims related to validity were unclear.

## Conclusion and future research

The aim of our study was to provide an overview of the existing literature related to characteristics of trustworthy management. We found that most of the studies explored leadership behaviours associated with trust in management. Leadership behaviours related to ethical leadership and caring for employees’ well-being were the most prominent in these studies. Based on our review, we present the following main suggestions for future research.

Firstly, based on the findings from the included studies, both leadership behaviours and organisational factors appear to be related to trust in management. However, these are not clearly distinct dimensions. For example, individual managers might positively or negatively influence employees’ perceptions of the work environment. Likewise, the work environment or organisational culture might influence individual leaders’ behaviours. Therefore, there is likely an interplay between factors in the work environment and individual leadership behaviours. More research is needed to untangle these relationships.

Secondly, we did not seek to explore whether certain leadership behaviours or organisational factors were more or less important in eliciting trust in management. The included studies did not explicitly aim to delineate such “hierarchies”. Future systematic review studies could explore possible causal relationships between leadership behaviours and organisational factors on employees’ trust in management.

Thirdly, the studies in our review explored characteristics of trustworthiness in formal managers. Informal leaders may also have a prominent role in some healthcare settings, but we cannot infer that the same characteristics will be relevant for understanding perceptions of trustworthiness in informal leaders. This is an aspect that could be researched further.

Lastly, only one study in our review reported results from two different samples (clinical and non-clinical workers). Future studies could investigate differences and similarities in how different employees in a medical setting (such as clinicians and non-clinicians) or healthcare professionals (such as nurses compared to physicians) view trustworthy management.

## Electronic supplementary material

Below is the link to the electronic supplementary material.

Additional file 1: Search strategies

Additional file 2: List of excluded papers and reasons

Additional file 3: Detailed summary of included studies

Additional file 4: Results from quantitative studies

Additional file 5: Results from qualitative studies

Additional file 6: PRISMA 2020 Checklist

Additional file 7: PRISMA 2020 Abstract Checklist

## Data Availability

All relevant data are within the paper and its attached Additional files.

## Abbreviations

OsloMet: Oslo Metropolitan University
UiO: University of Oslo
NICE: National Institute for Health and Care Excellence
EPI: Early Psychosis Intervention
SEM: structural equation modelling
