# Supplementary material for: Trustworthy management in hospital settings: a systematic review
Source: BMC Health Serv Res. 2023 Jun 20;23:662. doi: 10.1186/s12913-023-09610-5 (PMC10283186; doi:10.1186/s12913-023-09610-5)
Supplement: Supplementary file 2 — Additional file 2: List of excluded papers and reasons [file 12913_2023_9610_MOESM2_ESM.docx]

| **Additional file 2.** List of excluded papers and reasons for exclusion | | |
| --- | --- | --- |
| **Author(s) (Year)** | **Reasons for exclusion** | **Comments** |
| Azaare and Gross (1) | Trust was not an emergent theme |  |
| de Moura, Magalhães (2) | Trust was not an emergent theme |  |
| Denier, Dhaene (3) | Trust was not an emergent theme |  |
| Berghout, Fabbricotti (4) | Article is a systematic review |  |
| Denham (5) | No empirical data |  |
| Jønsson, Unterrainer (6) | Did not address study question |  |
| Norikoshi, Kobayashi (7) | Did not address study question |  |
| Rosengren and Bondas (8) | Did not address study question | Trust between managers was explored, not between managers and employees |
| Roseth, Austena (9) | Did not address study question | Explored managers’ trust in employees |
| Sripad, Ozawa (10) | Did not address study question | Explored trust from multiple perspectives and cannot distinguish between management and staff level |
| Teo, Lee (11) | Did not address study question |  |
| White (12) | Did not address study question |  |
| Bozaykut and Gurbuz (13) | Did not address study question | Trust as a tool, main focus was on power of doctor supervisor |
| Frost and Moussavi (14) | Did not address study question |  |
| Connell, Ferres (15) | Setting unclear (not a hospital) |  |
| Kaminskas, Bartkus (16) | Unclear description of trust |  |
| Hamlin (17) | Trust is not describe/explored |  |
| Katsaros, Tsirikas (18) | Trust is not describe/explored |  |

1. Azaare J, Gross J. The nature of leadership style in nursing management. British Journal of Nursing. 2011;20(11):672-80.

2. de Moura GMSS, Magalhães AMM, Souza, Agnol CMD. The social representations of the process of choosing leaders in the perspective of the nursing team. Revista da Escola de Enfermagem. 2012;46(5):1156-62.

3. Denier Y, Dhaene L, Gastmans C. "You can give them wings to fly': a qualitative study on values-based leadership in health care. Bmc Medical Ethics. 2019;20.

4. Berghout MA, Fabbricotti IN, Buljac-Samardzic M, Hilders C. Medical leaders or masters? - A systematic review of medical leadership in hospital settings. Plos One. 2017;12(9).

5. Denham CR. The 3 Ts of leadership engagement: Truth, trust, and teamwork. Journal of Patient Safety. 2006;2(3):162-70.

6. Jønsson TF, Unterrainer CM, Kähler HG. Do autonomous and trusting hospital employees generate, promote and implement more ideas? The role of distributed leadership agency. European Journal of Innovation Management. 2020.

7. Norikoshi K, Kobayashi T, Tabuchi K. A qualitative study on the attributes of nurses' workplace social capital in Japan. Journal of nursing management. 2018;26(1):74-81.

8. Rosengren K, Bondas T. Supporting "two-getherness": Assumption for nurse managers working in a shared leadership model. Intensive and Critical Care Nursing. 2010;26(5):288-95.

9. Roseth I, Austena H, Sommerseth E, Dahl B, Lyberg A, Bongaardt R. Fluid Boundaries and Moving Targets: Midwife Leaders' Perspectives on Continuing Professional Education. Sage Open. 2020;10(2).

10. Sripad P, Ozawa S, Merritt MW, Jennings L, Kerrigan D, Ndwiga C, et al. Exploring Meaning and Types of Trust in Maternity Care in Peri-Urban Kenya: A Qualitative Cross-Perspective Analysis. Qualitative Health Research. 2018;28(2):305-20.

11. Teo WL, Lee M, Lim W-S. The relational activation of resilience model: How leadership activates resilience in an organizational crisis. Journal of Contingencies and Crisis Management. 2017;25(3):136-47.

12. White HC. Some perceived behavior and attitudes of hospital employees under effective and ineffective supervisors. Journal of Nursing Administration. 1971;1(1):49-54.

13. Bozaykut T, Gurbuz FG. Power and trust in organizational relations: an empirical study in Turkish public hospitals. International Journal of Health Planning and Management. 2015;30(1):E1-E15.

14. Frost TF, Moussavi F. The relationship between leader power base and influence: The moderating role of trust. Journal of Applied Business Research (JABR). 1992;8(4):9-14.

15. Connell J, Ferres N, Travaglione T. Engendering trust in manager‐subordinate relationships. Personnel Review. 2003;32(5):569-87.

16. Kaminskas G, Bartkus EV, Pilinkus D. Leadership as Reciprocity of Leader and Followers. Inzinerine Ekonomika-Engineering Economics. 2011;22(2):175-85.

17. Hamlin RG. A study and comparative analysis of managerial and leadership effectiveness in the National Health Service: An empirical factor analytic study within an NHS Trust hospital. Health Services Management Research. 2002;15(4):245-63.

18. Katsaros KK, Tsirikas AN, Bani SMN. Exploring employees' perceptions, job-related attitudes and characteristics during a planned organizational change. International Journal of Business Science and Applied Management. 2014;9(1):36-50.
