# Supplementary material for: Trustworthy management in hospital settings: a systematic review
Source: BMC Health Serv Res. 2023 Jun 20;23:662. doi: 10.1186/s12913-023-09610-5 (PMC10283186; doi:10.1186/s12913-023-09610-5)
Supplement: Supplementary file 3 — Additional file 3: Detailed summary of included studies [file 12913_2023_9610_MOESM3_ESM.docx]

| **Additional file 3**. Detailed summary of included studies | | | |
| --- | --- | --- | --- |
| **Author(s) (Year)** | **Aim** | **Methodology, setting, participants and sample, instrument and measured concepts, data analysis** | **Country** |
| Araujo and Figueiredo (1) | To “identify the kind of work environment that should be offered by hospital leaders to their nursing staff in Brazil to generate job satisfaction, organizational commitment and organizational citizenship behaviour within their field of expertise.” (Abstract). | Quantitative. Setting: five private hospitals. Participants: nurses (N=171) and nurse technicians (N=274). Results are reported separately for each sample. Instrument: standardised questionnaire with 45 items on *internal climate dimensions* (*trust* being one of them) and 12 items on positive attitudes and behaviours, both categories rated on a five-point Likert scale. Data analysis: multivariate techniques factor analysis, multiple linear regressions. | Brazil |
| Bai, Lu (2) | To test “the impact of the morality and authoritarianism components of paternalistic leadership at different leader levels on employees’ personal initiative and affective trust.” (p.2). | Quantitative. Setting: 15 large-scale general hospitals. Participants: general employees (GE) (N=2365) and department leaders (DL) (N=270). Instrument: self-administered survey in which three concepts were measured on scales rated on a seven-point Likert scale. *Paternalistic leadership* was measured on a 12-item scale, and split between moral leadership and authoritarian leadership. GE rated their DLs paternalistic leadership, while DL rated their higher-up’s paternalistic leadership. GE self-reported *personal initiative* on a seven-item scale. And GE self-reported *affective trust* on a five-item scale. Data analysis: multilevel modelling analyses – hierarchical linear modelling. | China |
| Bobbio, Bellan (3) | “To test the impact of perceived empowerment leadership style expressed by nurse supervisor, nurses’ perceived organizational support, trust in the leader, and trust in the organization on nurses’ job burnout.” (Abstract). | Quantitative. Setting: a public general hospital. Participants: nursing staff (N=273). Instrument: self-administered questionnaire in which four concepts were measured on scales rated on a five-point Likert scale. *Empowering leadership* was assessed by a 38-item questionnaire spread over five dimensions (leading by example, participative decision making, coaching, informing and showing concern/interacting with the team). *Perceived organisational support* was evaluated by an eight-item survey. *Trust in leader and in organisation* were measured by a 12-item scale each, one for leaders and one for organisation. The scales were spread over three factors: keeping commitments, negotiating honestly and not taking excessive advantage. Lastly, *job burnout* was assessed on a 16-item survey grouped under three factors (emotional exhaustion, reduced professional accomplishment and cynicism). Data analysis: Pearson’s correlation coefficients were first used to test the nine hypotheses, and a path analysis using Linear Structural Relationships (LISREL) was undertaken to test the causal model and the mediation hypotheses. | Italy |
| Bobbio and Manganelli (4) | To “give support to and to further corroborate results in the literature linking perceived leadership style – and particularly servant leadership – perceived organizational support, trust in the leader and in the organization, job burnout among nurses and their subsequent intention to leave the hospital.” (Abstract). | Quantitative. Setting: two public general hospitals from two different regions. Participants: general nursing staff from the two hospitals (N1=371, N2=340). The results are reported separately for the two samples. Instrument: five concepts were measured by a self-administered questionnaire rated on a six-point Likert scale*. Servant leadership* was evaluated on a 30-item survey spread over eight dimensions (empowerment, accountability, standing back, humility, authenticity, courage, forgiveness and stewardship). *Perceived organisational support* was assessed by a three item scale.  *Trust in leader and in organisation* were measured by a 12-item scale each, one for leaders (nurse managers in this case) and one for organisation (the hospital). *Job burnout* was measured by a 16-item survey covering three factors (emotional exhaustion, professional efficacy and cynicism). And *intention to leave* the organisation was assessed by a three item scale. Data analysis: correlational analyses to test the first nine hypotheses, structural equation modelling to test the model and LISREL to test the 10^th^ mediation hypothesis. | Italy |
| Coxen, van der Vaart (5) | “To investigate the influence of authentic leadership on organizational citizenship behavior, through workplace trust among public health care employees” (Abstract). | Quantitative. Setting: 27 hospitals and/or clinics. Participants: Employees in different positions (administrative, management, specialist and other) (N=633). Instrument: self-administered questionnaire in which three constructs were measured. *Authentic leadership* evaluated subordinates’ perceptions of their leaders’ authenticity using a 14-item scale grouped under four dimensions (self-awareness, balanced processing, relational transparency and internalised moral perspective). The items were rated on a five-point Likert scale. *Workplace trust* was measured with a 32-item scale and comprised of trust in organisation, trust in immediate supervisor and trust in co-workers. The items were ranked on a four-point Likert scale. And *organisational citizenship behaviour* assessed assistance to co-workers (six questions) and assistance to organisation (three questions) on a six-point Likert scale. Data analysis: structural equation modelling. | South Africa |
| Cregård and Eriksson (6) | To explore the part-time physician-managers’ perception of the trust they receive from fellow physicians. (p. 282). | Qualitative. Setting: three hospitals. Participants: interviews with part-time physician-managers (N=8), nurse-managers (N=8), focus groups with nurse-managers and physician-managers (N=6) and a post-data saturation focus group with four nurse-managers and four physician-managers. Instrument: data collection focused on the three elements of trust: ability, benevolence and integrity. Interviewees were asked to give and describe example of situations pertaining to trust in physician-managers. The post-data saturation focus group addressed one main question: “What does diminished trust meant and does it matter?” (p.286). Data analysis: an analytical model based on theories of trust was used to analyse the data. | Sweden |
| (7) | To analyse “the impact of workplace empowerment and nursing staff’s perceptions of trustworthiness in their supervisor as determining factors of organizational commitment in a specific context of budget cuts.” (p. 703). | Quantitative. Setting: different hospital units. Participants: professionals (nurses, graduate nurses and specialized nurses) from various nursing services (N=189). Instrument: self-administered questionnaire in which three concepts were measured and rated on a five-point Likert scale. The prior *background of empowerment* was assessed using an 18-item questionnaire comprised of six factors (access to opportunity, access to information, support, access to resources, formal power and informal power). *Perceptions of trustworthiness in the supervisor* was measured on three dimensions (integrity, benevolence and ability) using an 18-item scale. And *organisational commitment* was evaluated on three dimensions (affective commitment, normative commitment and continuance commitment) using an 18-item scale. Data analysis: descriptive statistics and structural equation modelling techniques were employed | Portugal |
| Enwereuzor, Adeyemi (8) | To “investigate the relationship between ethical leadership and safety compliance, with trust in the leader as the mediator.” (Abstract). | Quantitative. Setting: 10 hospitals. Participants: staff nurses (N=237) completed a questionnaire sequentially in three time periods. Instrument: *ethical leadership* was measured on a 10-item one-dimensional scale, where respondents rated their agreement with statements regarding their ward/unit leader or immediate supervisor on a five-point scale. This concept along demographic variables were taken at T1. After two weeks, at T2, *trust in leader* was assessed by an eight-item one-dimensional scale, where items were rated on a seven-point response scale. At T3, after another two weeks, *safety compliance* was evaluated on an 11-item scale related to compliance with safety behaviours, where respondents indicate on a five-point scale how frequently engaged with the behaviour in the item. Data analysis: descriptive statistics, correlations, and ordinary least squares regression-based path analysis (using bootstrapped samples) were undertaken to test the hypotheses. | Nigeria |
| Fleig-Palmer, Rathert (9) | “To investigate the influence of health care managers’ informational and interpersonal mentoring behaviors on workers’ perceptions of their managers’ trustworthiness and the mediating role of trustworthiness on trust in the managers.” (Abstract). | Quantitative. Setting: an acute care hospital and associated clinics. Participants: clinical workers (registered nurses, certified nursing assistants, patient care assistants, respiratory therapists, pharmacists, phlebotomists, radiological technologists, drug/alcohol counsellors) and non-clinical workers (dietary, housekeeping, security, laundry, financial, managerial) (N=315). Instrument: respondents were provided with a definition of a mentor and asked to complete the survey with a mentor in mind, if they had one, or with their managers in mind. The survey addressed three concepts ranked on a five-point Likert scale. The *mentoring function* was evaluated by a 27-item scale, split into informational mentoring, such as exposure and visibility, coaching, sponsorship, challenging assignments and interpersonal mentoring representing counselling, role modelling, acceptance and confirmation, friendship, protection. The *trustworthiness factors* of ability, integrity and benevolence were measured on an eight-item scale. And *trust* was assessed by a seven-item scale. Control variables (educational level and tenure) were also gathered. Data analysis: common method variance analysis. | USA |
| Freysteinson, Celia (10) | “To understand nursing leaders’ experiences during the pandemic.” (Abstract). | Qualitative. Setting: 11 hospitals in a large health care centre. Participants: individuals with leadership experience (N=28) were interviewed, such as clinical managers, managers, directors, chief nursing officers (CNO) and assistant vice president (AVP). Instrument: semi-structured interview questions related to nursing leaders’ feelings, decisions, motives and self-talk during a crisis (pandemic). Data analysis: naïve reading, structural explanation and phenomenological interpretation. | USA |
| Laschinger, Finegan (11) | “To test a model linking staff nurses’ workplace empowerment, organizational trust, and organizational commitment.” (p. 414). | Quantitative. Setting: urban tertiary care hospitals. Participants: nurses (N=412). Instrument: surveys were mailed with a three-week follow-up. Five concepts were measured. *Work empowerment* was measured by four subscales that assessed nurses’ perception of access to opportunity, information, support and resources. *Formal power* in the work environment was evaluated by a three-item scale that measured nurses’ perception of job flexibility, visibility and recognition. *Informal power* was assessed by another three-item scale that measured nurses’ perception of sponsor support, peer networking and professional relationships. *Interpersonal trust at work* was evaluated by a 12-item survey with four subscales that measured faith in the intentions of peers and managers; and confidence in actions of peers and managers. From these subscales, the authors combined those related to managers and created a six-item measure of *organisational trust*. And *affective and continuance organisational commitment* were measured each by six-item subscales. Data analysis: structural equation modelling. | Canada |
| McCabe and Sambrook (12) | “To explore the antecedents, attributes and consequences of the concept of trust among nursing professionals, at individual, interpersonal and organizational levels” (p. 818). | Qualitative. Setting: two hospitals (a large acute organization and a small community organisation). Participants: nurses from the acute (N=12) and community (N=16) hospitals, and nurse managers from the acute (N=8) and community hospitals (N=3) were interviewed. Instrument: semi-structured, open-ended questions related to “(*1)* how participants conceptualized trust and the level of trust within their working environment, *(2)* the characteristics and attributes of trust and trustworthy managers and *(3)* the consequences of low trust” (p. 819). Data analysis: concept analysis, thematic analysis. | UK |
| Simha and Stachowicz-Stanusch (13) | “To investigate the effects of ethical climates on two facets of trust, namely, trust in supervisors and trust in organization” (p. 24). | Quantitative. Setting: seven hospitals. Participants: hospital administrators and management personnel (N=178). Instrument: *ethical climate* was assessed using a 12-item survey, split in three aspects (egoistic-local climates, benevolent-local climates and principles-local climates); with responses given on a six-point Likert scale. *Trust in supervisor* was evaluated by an eight-item scale, responses being given on a five-point Likert scale. And *trust in organisations* was measured using a 4-item scale, responses being given on a five-point Likert scale. Data analysis: factor analyses and structural equation modelling. | Poland |
| Stander, de Beer (14) | Firstly, to establish whether the authentic leadership style “could predict optimism, trust in the organisation and work engagement” (Abstract) among employees in various positions in public hospitals and clinics. Secondly, to determine whether optimism and trust in the organisation could mediate the relationship between AL [authentic leadership] and work engagement.” (Abstract). | Quantitative. Setting: 27 public hospitals and clinics. Participants: participants (N_total_=633) in management positions (N=94), specialist positions (N=65), administrative positions (N=106) and employees in other positions (N=275) completed a questionnaire. Instrument: five concepts were measured alongside demographic characteristics. *Authentic leadership* was evaluated by a 14-item survey scored on a five-point Likert scale. *Job resources* were measured by a 10-item survey, with two dimensions (relationship with colleagues and communication), scored on a frequency scale. *Optimism* was assessed by a 12-item questionnaire scored on a six-point Likert scale. *Trust in the organisation* was measured through a nine-item survey scored on a seven-point Likert scale. And *work engagement* was evaluated by an eight-item questionnaire rated on a seven-point frequency scale. Data analysis: structural equation modelling. | South Africa |
| Stasiulis, Gibson (15) | “To examine how trust within an EPI [early psychosis intervention] setting is produced and operates” (Abstract). | Qualitative. Setting: An EPI clinic. Participants: clinic staff comprised of administrative/research workers (N=5), service coordinators (N=4), occupational therapists (N=2), peer support workers (N=2), family support workers (N=2), drug counsellor (N=1), recreation therapist (N=1), psychiatrist (N=1), young people attending the clinic (N=4) and family members (N=5) were interviewed. In addition, participant observations and text reviews of clinic documents were conducted. Instrument: the in-depth interviews were guided by “questions that focused on obtaining accounts of participants’ everyday activities in the clinic” (p.3). Data analysis: reading and mapping. | Canada |
| Topp and Chipukuma (16) | “To examine the relevance of and factors contributing to the production of trust, and related, the influence of trust (or its absence) on the quality and responsiveness of service delivery in a low-resource setting.” (p. 194). | Qualitative. Setting: four primary health centres, which include outpatient and inpatient departments. Participants: health care workers (N=60) participated in in-depth interviews, key-informants (N=14) comprised of government and nongovernment officials were interviewed. Patients (N=180) also participated in semi-structured interviews. In addition, facility audits, unstructured observations and research memos as well as structured observations were performed. Instrument: to avoid risk of social desirability, the term “trust” was not explicitly asked about; rather the questions posed in the interviews were aimed “to elicit detailed descriptions of interactions among and between staff and patients to provide insight into whether and why trust may be present in certain relationships” (p.195). Several themes were explored. Data analysis: deductive and inductive analysis. | Zambia |
| Weaver, Lindgren (17) | This focused ethnographic study was conducted in two parts. The aim of the first part was to obtain “the staff nurses’ perception of the supervisors’ role”, while the second aimed to “explore the supervisors’ perspective on practices used to enhance safety.” (Abstract). | Qualitative. Setting: 37 hospitals in different regions. Participants: in part one, evening-night-weekend staff nurses (N=39) participated in seven focus groups. While in part two, administrative supervisors (N=30) participated in semi-structured telephone interviews. Instrument: the participants were asked about the practices of administrative supervisors. Data analysis: an inductive, systematic and iterative analysis strategy was employed. | USA |
| Wong and Cummings (18) | “To test a model linking authentic leadership behaviors with trust in management, perceptions of supportive groups and work outcomes using a health care employee dataset and structural equation modelling procedures.” (p.7). | Quantitative. Setting: 17 cancer treatment facilities. Participants: clinical care providers (N=147) such as nurses, pharmacists, physicians and other professionals; and nonmedical employees (N=188) such as administrative, support and research staff completed a survey. Results were reported separately for each sample. Instrument: several variables were measured which then served to develop latent concepts that were used in the theoretical model. *Leadership practices* of immediate supervisors was evaluated by a 30-item tool comprised of five aspects (challenging the process, modelling the way, inspiring a shared vision, enabling others to act and encouraging the heart). From these, the latent concept of ***leadership behaviour*** was created and is comprised of seven behaviours (self-awareness, relational transparency, balanced processing, ethical behaviour, trustworthiness, supportiveness and empowering). *Perceptions of work-life* were measured by a 29-item scale over six areas (workload, control, reward, community, fairness and values). From these items, the latent concepts of ***trust in management*** and ***supportive group*** were created. *Emotional health and well-being* of staff were assessed by a 16-item questionnaire grouped under three subscales (emotional exhaustion, cynicism and professional efficacy). From these items, alongside items from the perceptions of work-life, the latent concepts of ***voice, performance*** and ***burnout*** were developed. Data analysis: structural equation modelling. | Canada |

1. Araujo CAS, Figueiredo KF. Brazilian nursing professionals: leadership to generate positive attitudes and behaviours. Leadership in health services (Bradford, England). 2019;32(1):18-36.

2. Bai S, Lu F, Liu D. Subordinates' responses to paternalistic leadership according to leader level. Social Behavior and Personality: An International Journal. 2019;47(11):1-14.

3. Bobbio A, Bellan M, Manganelli AM. Empowering leadership, perceived organizational support, trust, and job burnout for nurses: A study in an Italian general hospital. Health Care Management Review. 2012;37(1):77-87.

4. Bobbio A, Manganelli AM. Antecedents of hospital nurses' intention to leave the organization: A cross sectional survey. International Journal of Nursing Studies. 2015;52(7):1180-92.

5. Coxen L, van der Vaart L, Stander MW. Authentic leadership and organisational citizenship behaviour in the public health care sector: The role of workplace trust. 2016. 2016;42(1).

6. Cregård A, Eriksson N. Perceptions of trust in physician-managers. Leadersh Health Serv (Bradf Engl). 2015;28(4):281-97.

7. da Costa Freire CMF, Azevedo RMM. Empowering and trustful leadership: Impact on nurses' commitment. Personnel Review. 2015;44(5):702-19.

8. Enwereuzor IK, Adeyemi BA, Onyishi IE. Trust in leader as a pathway between ethical leadership and safety compliance. Leadership in Health Services. 2020;33(2):201-19.

9. Fleig-Palmer MM, Rathert C, Porter TH. Building trust: The influence of mentoring behaviors on perceptions of health care managers' trustworthiness. Health Care Management Review. 2018;43(1):69-78.

10. Freysteinson WM, Celia T, Gilroy H, Gonzalez K. The Experience of Nursing Leadership in a Crisis: A Hermeneutic Phenomenological Study. Journal of nursing management. 2021;19.

11. Laschinger HKS, Finegan J, Shamian J, Casier S. Organizational Trust and Empowerment in Restructured Healthcare Settings: Effects on Staff Nurse Commitment. JONA: The Journal of Nursing Administration. 2000;30(9):413-25.

12. McCabe TJ, Sambrook S. The antecedents, attributes and consequences of trust among nurses and nurse managers: A concept analysis. International Journal of Nursing Studies. 2014;51(5):815-27.

13. Simha A, Stachowicz-Stanusch A. The effects of ethical climates on trust in supervisor and trust in organization in a Polish context. Management Decision. 2015;53(1):24-39.

14. Stander FW, de Beer LT, Stander MW. Authentic leadership as a source of optimism, trust in the organisation and work engagement in the public health care sector. Sa Journal of Human Resource Management. 2015;13(1).

15. Stasiulis E, Gibson BE, Webster F, Boydell KM. Resisting governance and the production of trust in early psychosis intervention. Social Science & Medicine. 2020;253.

16. Topp SM, Chipukuma JM. A qualitative study of the role of workplace and interpersonal trust in shaping service quality and responsiveness in Zambian primary health centres. Health Policy Plan. 2016;31(2):192-204.

17. Weaver SH, Lindgren TG, Cadmus E, Flynn L, Thomas-Hawkins C. Report From the Night Shift: How Administrative Supervisors Achieve Nurse and Patient Safety. Nursing administration quarterly. 2017;41(4):328-36.

18. Wong CA, Cummings GG. The influence of authentic leadership behaviors on trust and work outcomes of health care staff. Journal of Leadership Studies. 2009;3(2):6-23.
