# Supplementary material for: Trustworthy management in hospital settings: a systematic review
Source: BMC Health Serv Res. 2023 Jun 20;23:662. doi: 10.1186/s12913-023-09610-5 (PMC10283186; doi:10.1186/s12913-023-09610-5)
Supplement: Supplementary file 4 — Additional file 4: Results from quantitative studies [file 12913_2023_9610_MOESM4_ESM.docx]

| **Additional file 4.** Results from quantitative studies | |
| --- | --- |
| **Author(s) (Year)** | **Results related to trust extracted from the quantitative studies included in the review.** |
| Araujo and Figueiredo (1) | One broad hypothesis was tested in this study and an analytical model was proposed. “H1: Positive attitudes and behaviors from nursing professionals may be explained by the internal climate dimensions for these professionals.” [partially confirmed]. *Trust* was one of the nine dimensions of internal climate resulted from the factor analysis and was measured over five dimensions (“the superiors make my job easier”, “the superiors care about my well-being at work”, “my superiors are accessible and open to dialogue”, “the nursing team participates of the hospital decisions” and “my individual rights are respected” Table II). From the measured variables of positive attitudes and behaviours, a Positive Consequence Index (PC index) was obtained, on three dimensions: job satisfaction, affective commitment and organisational citizenship behaviour. From the multiple linear regression analysis, trust, alongside other six dimensions, turned out to be statistically significant in explaining 41.20% of the total variation of the PC Index for nurses. |
| Bai, Lu (2) | Trust was conceptualised as employees’ affective trust in their direct leaders. Trust was positively related to direct leaders’ moral leadership and negatively related to direct leaders’ authoritarian leadership. |
| Bobbio, Bellan (3) | Empowering leadership was comprised of five dimensions (leading by example, participative decision making, coaching, informing, showing concern/interacting with the team), and all were positively correlated with trust in the leader and trust in the organisation, the relationship being statistically significant.  Trust in the leader and trust in the organisation were also positively correlated.  Additionally, a causal model was tested, which revealed that trust in leader was increased by two empowering leadership factors (leading by example and showing concern/interacting with the team); and that trust in the organisation was increased by one factor of empowering leadership (informing) and perceived organisational support. |
| Bobbio and Manganelli (4) | Servant leadership and perceived organisational support were both positively correlated to trust in the leader and trust in the organisation.  Also, trust in the leader and trust in the organisation were positively correlated to each other. |
| Coxen, van der Vaart (5) | Authentic leadership was a significant predictor of workplace trust across three dimensions (organisation, supervisor and co-worker). Specifically, authentic leadership explained 36%, 67% and 23% of the variance in trust in the organisation, in the immediate supervisor and in co-worker respectively. Although authentic leadership is comprised of four dimensions in the literature (self-awareness, relational transparency, balanced processing and moral perspective), for the model to have a good fit, the authors decided to use authentic leadership as a one-factor structure (conceptualised as an overarching leadership style). |
| da Costa Freire and Azevedo (6) | An empowering work context (characterised by formal power, informal power, resources, opportunity, support and information) significantly predicted nurses’ perceptions of their supervisor’s trustworthiness (measured with separate items for integrity, benevolence and ability). |
| Enwereuzor, Adeyemi (7) | Based on a path analysis, the author found that ethical leadership had a positive relationship with trust in a leader. |
| Fleig-Palmer, Rathert (8) | Two hypotheses were tested, both relevant to trust.  “H1. Ability will mediate the relationship between informational mentoring behaviors and trust.” (p.71) [confirmed].  “H2. Integrity and benevolence will mediate the relationship between interpersonal mentoring behaviors and trust.” (p. 72) [confirmed]. |
| Laschinger, Finegan (9) | Two models were tested using structural equation modelling, but relevant to this paper were the findings of the second model which state that trust in management was significantly associated with the empowerment factors: access to opportunity, resources, information and support; the last two having the strongest relationship. |
| Simha and Stachowicz-Stanusch (10) | The authors tested different hypotheses related to organisational ethical climates and trust. They were all supported.  Egoistic-local climates were negatively related to trust in supervisor and trust in organisation.  Benevolent-local climates were positively related to trust in supervisor and trust in organisation. |
| Stander, de Beer (11) | The authors found that authentic leadership positively predicted trust in the organization. |
| Wong and Cummings (12) | Results are reported separately for the two groups, clinical care providers and non-clinical employees. The results of the structural equation modelling for the clinical sample show no direct significant effects between leadership behaviours and trust in management (p.14). One leadership behaviour, supportiveness, had a significant indirect effect on trust in management through the variable supportive group (p.14). For the non-clinical sample, one leadership behaviour, relational transparency, had a direct and significant influence on trust in management (p. 16). |

1. Araujo CAS, Figueiredo KF. Brazilian nursing professionals: leadership to generate positive attitudes and behaviours. Leadership in health services (Bradford, England). 2019;32(1):18-36.

2. Bai S, Lu F, Liu D. Subordinates' responses to paternalistic leadership according to leader level. Social Behavior and Personality: An International Journal. 2019;47(11):1-14.

3. Bobbio A, Bellan M, Manganelli AM. Empowering leadership, perceived organizational support, trust, and job burnout for nurses: A study in an Italian general hospital. Health Care Management Review. 2012;37(1):77-87.

4. Bobbio A, Manganelli AM. Antecedents of hospital nurses' intention to leave the organization: A cross sectional survey. International Journal of Nursing Studies. 2015;52(7):1180-92.

5. Coxen L, van der Vaart L, Stander MW. Authentic leadership and organisational citizenship behaviour in the public health care sector: The role of workplace trust. 2016. 2016;42(1).

6. da Costa Freire CMF, Azevedo RMM. Empowering and trustful leadership: Impact on nurses' commitment. Personnel Review. 2015;44(5):702-19.

7. Enwereuzor IK, Adeyemi BA, Onyishi IE. Trust in leader as a pathway between ethical leadership and safety compliance. Leadership in Health Services. 2020;33(2):201-19.

8. Fleig-Palmer MM, Rathert C, Porter TH. Building trust: The influence of mentoring behaviors on perceptions of health care managers' trustworthiness. Health Care Management Review. 2018;43(1):69-78.

9. Laschinger HKS, Finegan J, Shamian J, Casier S. Organizational Trust and Empowerment in Restructured Healthcare Settings: Effects on Staff Nurse Commitment. JONA: The Journal of Nursing Administration. 2000;30(9):413-25.

10. Simha A, Stachowicz-Stanusch A. The effects of ethical climates on trust in supervisor and trust in organization in a Polish context. Management Decision. 2015;53(1):24-39.

11. Stander FW, de Beer LT, Stander MW. Authentic leadership as a source of optimism, trust in the organisation and work engagement in the public health care sector. Sa Journal of Human Resource Management. 2015;13(1).

12. Wong CA, Cummings GG. The influence of authentic leadership behaviors on trust and work outcomes of health care staff. Journal of Leadership Studies. 2009;3(2):6-23.
