# Supplementary material for: Trustworthy management in hospital settings: a systematic review
Source: BMC Health Serv Res. 2023 Jun 20;23:662. doi: 10.1186/s12913-023-09610-5 (PMC10283186; doi:10.1186/s12913-023-09610-5)
Supplement: Supplementary file 5 — Additional file 5: Results from qualitative studies [file 12913_2023_9610_MOESM5_ESM.docx]

| **Additional file 5**. Results from qualitative studies | |
| --- | --- |
| **Author(s) (Year)** | **Results related to trust extracted from the qualitative studies included in the review** |
| Cregård and Eriksson (1) | The findings are grounded on the perceptions of the respondents (physician- and nurse-managers) of perceptions of physicians and are grouped into reasons that increase and decrease trust in part-time physicians for each element of trust (ability, benevolence and integrity) (Table 1, p.287). In terms of *ability*, the reason for increased trust is related to physician-manager’s medical competence and how this is valuable in making managerial health care decisions. While reasons for decreased trust arise from: (1) the dual responsibility physician-managers have over both managerial and medical issues, which can lead to conflicts; (2) staff and financial issues that ask for a full-time commitment and (3) that physician-managers should have extensive involvement in medical practice in order to maintain competence in daily medical work. In regards to *benevolence*, increased trust resides in physician-managers that show care for patients, colleagues and other health care professionals. Trust can decrease when: (1) physician-managers might show less importance to principles of equity and ethics when budget constraints are considered; and when (2) collegiality among the medical staff is downplayed by physician-managers. Lastly, under the element of *integrity*, trust is strengthened by physician-managers’ understanding of health care issues from different perspectives. Reasons for decreased trust arise from: (1) physician-managers’ inability to prioritise both managerial and medical issues and (2) the concern that physician-managers may not be able to fulfil professional demands.  It was noted that the difficulty of combining the managerial and medical roles in one position is a shared feature of the reasons for decrease in perceived trust (p.292). |
| Freysteinson, Celia (2) | “Trusting and earning trust” was one of five phenomenological themes that emerged from the study. Under this theme, the authors mention that leaders became aware of how face-to-face interaction were crucial to earing the trust of the employees, and that leaders got a sense that being transparent increased trust. |
| McCabe and Sambrook (3) | The authors identified three main themes of trust: *antecedents, attributes and consequences* of trust; only first two being relevant to this paper.  *Antecedents* of trust converged mainly on organisational factors: immediate work environment, communication systems and new management practices taken from the private sector. The latter could decrease perceptions of trust.  *Attributes* of trust focused on individual factors: *leadership, professionalism, communication styles and confidentiality*. Individuals that led by example and showcased good judgement were more likely to gain trust. Managers who were considered accessible, approachable, hands on and involved were more likely to be trusted by nurses, as these characteristics instilled a sense of support, respect and value in nurses. The opposite was true for managers that were “perceived as ‘inaccessible’, ‘removed’ or those managers higher up within the organisational hierarchy”. Communication styles characterised by openness, honesty and clarity were considered as both attributes, antecedent and consequence of trust. *Confidentiality* *and discretion* were linked to the communication, and were noted as important for a good relationship with colleagues and line-managers. ‘Professional competence’, ‘consistency’, ‘accountability’ and ‘objectivity’ in decision-making and behavior were viewed as attributes of ‘trusted’ line-managers and nurses”. |
| Stasiulis, Gibson (4) | To outline the development of trust at an early psychosis intervention (EPI) clinic, several themes were established*: (1)* *disjuncture in governance and acts of resistance, (2) facilitating trust work: enacting reflective practices*, *(3)* *facilitating trust work: working groups*, *(4)* *designing protocols that generated trust*, *(5)* *young people and family member’s trust* and *(6) protocols containing regulatory functions*.  For the first theme, tensions surfaced as there were competing objectives, since the EPI clinic was affiliated and subject to an academic hospital’s accountability and quality control criteria. To alleviate the tensions, the clinic manager alongside staff engaged in acts of resistance. The manager’s efforts to guard the autonomy of the staff and lighten their work, contributed to the staff’s feelings of care and respect, which the authors label as elements of trust (p. 4).  The study findings showed that, “the two main factors that set out the foundation for building trust were the clinic manager’s efforts: to circumvent hospital rulings that hindered service providers’ EPI work; and to implement reflective leadership practices that aimed to promote an organizational environment of trust” (p. 6). The clinic manager engaged in reflective leadership practices by providing opportunities for staff to reflect on and share issues, by including staff in making decisions where possible and listening to their input (p. 4-5). |
| Topp and Chipukuma (5) | From the interviews, a range of factors contributing to generally weak trust in employer, supervisor and colleagues was identified. (Table 2, page 197). For trust in employer, four common themes (factors) were uncovered: *inadequate or delayed salary*, as well as insufficient pay reported by many providers, *shortages in staff and increased workload* associated with the shortages, *sub-optimal working and/or environmental conditions*, such as drug and equipment shortages, and *inadequate administrative and supervisory support* (p. 197)*.* In regards to trust in supervisor, three common factors emerged that contributed to weak trust: providers’ perception that those in charge of *overall or departmental sites were unfair or inconsistent*, for example when selecting staff for workshops or trainings; perceived *lack of problem-solving capacity*, such as addressing material shortages; and *poor communication and information dissemination* (p.198). Under the umbrella of trust in colleagues, two themes emerged: perceived *lack of accountability* if staff in “other” departments and the perception that *providers working in “different departments were somehow advantaged*” (p.198). |
| Weaver, Lindgren (6) | The overarching theme relates to the administrative supervisors being the hospital leaders whose goal is to make it safely through the shit (evening, night or weekend) (p.330). The components of this theme are represented by what the supervisors do to reach the goal *“make it work”* and the results of those actions, *“outcome”*. The sub-themes under “make it work” are: establishing trust, doing rounds, educating, providing support and the sub-themes under “outcome” are: nurse safety and patient safety. Supervisors purposefully worked to establish trust with the staff, and from one supervisor’s perspective, this process was made easier for those who were friendly, personable and “a people person” (p.331). The authors also mention that supervisors who were unapproachable were not trusted by the staff nurses. The importance of an approachable, friendly and honest supervisor that has clinical expertise was also confirmed by staff nurses (p. 331). Although establishing trust is the direct process to attain that relationship between supervisors and staff, the authors mention that the other three process (doing rounds, education and providing support) also contribute to developing trust (p.331). |

1. Cregård A, Eriksson N. Perceptions of trust in physician-managers. Leadersh Health Serv (Bradf Engl). 2015;28(4):281-97.

2. Freysteinson WM, Celia T, Gilroy H, Gonzalez K. The Experience of Nursing Leadership in a Crisis: A Hermeneutic Phenomenological Study. Journal of nursing management. 2021;19.

3. McCabe TJ, Sambrook S. The antecedents, attributes and consequences of trust among nurses and nurse managers: A concept analysis. International Journal of Nursing Studies. 2014;51(5):815-27.

4. Stasiulis E, Gibson BE, Webster F, Boydell KM. Resisting governance and the production of trust in early psychosis intervention. Social Science & Medicine. 2020;253.

5. Topp SM, Chipukuma JM. A qualitative study of the role of workplace and interpersonal trust in shaping service quality and responsiveness in Zambian primary health centres. Health Policy Plan. 2016;31(2):192-204.

6. Weaver SH, Lindgren TG, Cadmus E, Flynn L, Thomas-Hawkins C. Report From the Night Shift: How Administrative Supervisors Achieve Nurse and Patient Safety. Nursing administration quarterly. 2017;41(4):328-36.
